# *In Vitro* and *In Vivo* Toxicity of *Garcinia* or Hydroxycitric Acid: A Review

**DOI:** 10.1155/2012/197920

**Published:** 2012-08-09

**Authors:** Li Oon Chuah, Swee Keong Yeap, Wan Yong Ho, Boon Kee Beh, Noorjahan Banu Alitheen

**Affiliations:** ^1^School of Industrial Technology, University Science Malaysia, 11800 Penang, Malaysia; ^2^Institute of Bioscience, University Putra Malaysia, 43400 Serdang, Selangor, Malaysia; ^3^Department of Cell and Molecular Biology, Faculty of Biotechnology and Biomolecular Sciences, Universiti Putra Malaysia, 43400 Serdang, Selangor, Malaysia; ^4^Department of Bioprocess Technology, Faculty of Biotechnology and Biomolecular Sciences, University Putra Malaysia, 43400 Serdang, Selangor, Malaysia

## Abstract

Obesity is one of the pandemic chronic diseases commonly associated with health disorders such as heart attack, high blood pressure, diabetes or even cancer. Among the current natural products for obesity and weight control, *Garcinia* or more specifically hydroxycitric acid (HCA) extracted from *Garcinia* has been widely used. The evaluation of the potential toxicity of weight control supplement is of the utmost importance as it requires long term continuous consumption in order to maintain its effects. Majority of reports demonstrated the efficacy of *Garcinia*/HCA without any toxicity found. However, a few clinical toxicity reports on weight-loss diet supplements of which some were combinations that included *Garcinia*/HCA as an active ingredient showed potential toxicity towards spermatogenesis. Nonetheless, it cannot be concluded that *Garcinia*/HCA is unsafe. Those products which have been reported to possess adverse effects are either polyherbal or multi-component in nature. To date, there is no case study or report showing the direct adverse effect of HCA. The structure, mechanism of action, long history of the use of *Garcinia*/HCA and comprehensive scientific evidence had shown “no observed adverse effect level (NOAEL)” at levels up to 2800 mg/day, suggesting its safety for use.

## 1. Introduction

Focus on disease prevention by complementary supplementation of nutraceutical products to medication has heralded the growing demand for healthy food. In addition, rising healthcare costs have greatly boosted the growth of the nutraceutical industry [1]. Nonetheless, scientific evidence confirming the effects claimed by the nutraceutical products is scanty at best. Scientific investigation on potential health promoting effects of herbal preparations as diet supplement is essential for new discoveries of alternative therapies. The consistency, safety, and bioavailability of the active herbal supplements are of utmost importance [2]. Taking these into considerations, the 103rd US Congress passed the Dietary Supplement Health and Education Act (DSHEA) in 1994 [3, 4]. In the “findings” section of the Act, the US Congress noted that “consumers should be empowered to make choices about preventive health care programs based on data from scientific studies of health benefits related to particular dietary supplements” [5]. Phenomenal growth in consumer acceptance of dietary supplements was evidenced by the 10 million daily users and an estimated annual market of $12 billion, in the 4.5 years after the passage of the DSHEA [6]. Currently, the dietary supplement industry in the USA is fully regulated under DSHEA, Food and Drug Administration (FDA) Modernization Acts of 1997 and 2011, Dietary Supplement and Nonprescription Drug Consumer Protection Act (DSNDCPA) of 2006 and other acts of US Congress, which provides the US FDA with statutory authority on regulation of the industry [4].

*Garcinia *has been used for centuries in Asian countries for culinary purposes as a condiment and flavoring agent in place of tamarind or lemon, and to make meals more filling. *Garcinia* or more specifically *G. cambogia*, *G. atroviridis,* and *G. indica* have been found to contain large amounts of hydroxycitric acid (HCA) [7]. It has been widely used as an antiobesity herbal supplement for decades around the world. This is because HCA is able to inhibit lipogenesis, a process in which carbohydrate is converted to fat in the body, via the inhibition of ATP citrate lyase (EC 4.1.3.8) in cells [8, 9]. Prevention of carbohydrate conversion to fat by HCA thus induces the body to oxidize the excess carbohydrates, promoting glycogen storage, which in turn may play a part in suppressing the appetite [10]. Furthermore, HCA suppressed the feeling of hunger by increasing the release/availability of serotonin, a neurotransmitter that regulates eating behavior and appetite control [11, 12]. It had also been reported that HCA decreased serum leptin in mice [13] and human [14], as well as expression level of abdominal fat leptin in rats [15]. Despite many publications on *Garcinia/*HCA exerting antiobesity effects, the results on the effects of HCA on appetite, body weight and energy expenditure (EE), and its potential contribution as a weight loss agent in humans were controversial [16–19]. Although some mild adverse effects such as headache, and upper respiratory tract and gastrointestinal symptoms have been reported in overweight subjects, the nonsignificant difference in the occurrence of the adverse effects between treatment groups [20] did not justify the definitive risk of HCA consumption. In the studies published thus far, the safety and efficacy of HCA have been the subject of debate. The aim of this review was to critically assess the evidence from the *in vitro, in vivo,* and clinical trials on the safety of *Garcinia/*HCA as a dietary supplement for treating obesity.

## 2. *In Vitro *and *In Vivo* Toxicology Studies 

### 2.1. Cytotoxicity

Varalakshmi et al. [21] evaluated the *in vitro* antiproliferative effects of the aqueous extracts of dried fruit rind of *G. indica* (0, 50, 100, 200 *μ*g/mL) on Balb/c 3T3 mouse fibroblasts and human peripheral lymphocytes. The results showed that *G. indica* extracts inhibited lymphocytes and 3T3 fibroblast cell survival. Thus, the authors concluded that *G. indica* extracts exhibited pronounced cytotoxic effects. However, there was a flaw in their methodology, since the authors also reported that *Azadirachta indica* and *Coleus aromaticus* exhibited cytotoxic effects on lymphocytes despite the low cell viability in the control group (only 50–55% of viable lymphocytes). In the case of *G. indica*, percentage of viability in lymphocytes was not even mentioned. Thus, definitive conclusion of *G. indica* induced cytotoxicity could not be drawn due to the poorly-described methodology of their study.

### 2.2. Genotoxicity

K. H. H. HHLee and B. M. Lee [22] conducted a study to evaluate the genotoxicity of Super CitriMax (HCA-SX) containing 60% HCA using bacterial reverse mutation assay (Ames test), *in vitro* chromosomal aberration (CA) test, and *in vivo* micronucleus (MN) test. For the Ames test (plate incorporation method), five *Salmonella typhimurium *strains (TA98, TA100, TA102, TA1535, and TA1537) were used and six different doses of HCA-SX (0, 20, 100, 500, 2500, 12500 *μ*M/plate) were tested. No significant increase (*P* < 0.05) in the number of revertants was observed, indicating that HCA-SX did not induce mutagenic activity in any of the five bacterial strains tested, under any of the activation conditions examined. In the CA test, HCA-SX-treated Chinese hamster ovary cells were fixed on glass slides and stained with Giemsa staining solution. The stained cells were viewed under an optical microscope, where at least 100 metaphases were counted at a resolution of 1000x. No significant mutagenic potency was detected by the CA tests. In the MN test, suspensions containing HCA-SX were administered to 7-to-8-week-old old ICR mice via intraperitoneal (ip) injection as follows: group 1, negative control (vehicle alone); group 2, positive control (Mitomycin C, 2 mg/kg); groups 3, 4, 5, 6, and 7, HCA-SX-treated (at dose levels of 20, 100, 500, 2500, or 12,500 *μ*mol/kg, resp.). The bone marrow cells were fixed, stained, and viewed using an optical microscope. HCA tended to increase the number of micronucleated polychromatic erythrocytes (MNPCEs/1000 polychromatic erythrocytes) and the polychromatic erythrocytes/normochromatic erythrocytes PCE/(PCE + NCE) ratios, and they reached significance level at a dose of 12,500 *μ*mol/kg. Taken all together, the authors suggested that HCA-SX possessed no genotoxic effect by bacterial or by chromosome aberration testing, but preferentially induced micronuclei.

However, Lau et al. [23] refuted the authors' conclusion in the abstract section that the “results suggest that HCA preferentially induces micronuclei” [22]. Considering that DMSO may react with HCA-SX to induce adverse effects, they suggested that the highest dose used in the study (12,500 *μ*mol/kg) may have been too high and exceeded the maximum tolerated dose. Besides, Lau and colleagues pointed out several limitations in the experimental design and the interpretation of the results, as follows: (1) selection of ip delivery of HCA-SX rather than the recommended oral administration for this supplement, (2) selection of DMSO as a vehicle, which was not recommended for the *in vivo* rodent erythrocyte micronucleus assay, (3) five different HCA-SX doses were selected in the absence of a prior ip LD50 determination, (4) the range of doses (increased by a factor of 5) chosen in the study deviated from that of the conventional dose levels used in toxicological studies, (5) no significant difference (*P* < 0.05) in the values of percent MNPCE between 500 *μ*mol/kg and 12,500 *μ*mol/kg, suggesting the use of the highest dose was probably unnecessary, and (7) poor statistical analysis.

### 2.3. Acute and Short-Term Toxicological Studies

Acute safety studies of HCA-SX (containing 60% HCA) as demonstrated in acute oral and dermal toxicity studies were conducted [11, 12]. In the acute oral toxicological study, the acute oral median lethal dose (LD_50_) was determined to evaluate the potential systemic toxicity of HCA-SX when administered as a single dose to male and female Albino rats. HCA-SX at a single dose of 5000 mg/kg was administered orally via gastric intubation in a dose volume of 10 mL/kg. Toxicological studies revealed no death, remarkable body weight changes or gross necropsy findings in Albino rats following a single oral dose of 5000 mg/kg, equivalent to 350 g or 233 times the maximum dose of 1.5 g/day of HCA in humans. Clinical findings were limited to soft stool and rales for one male and two female rats, respectively. Taken all together, the authors suggested that the oral LD_50_ of HCA-SX in rats (administered once orally via gastric intubation to fasted male and female Albino rats) was more than 5000 mg/kg [12].

### 2.4. Subchronic and Chronic Toxicological Studies

However, a long-term study on the safety and efficacy of HCA-SX or any of the HCA products still remained to be conducted. Hence, Shara et al. [24, 25] extended their study and conducted a 90-day chronic safety study in both male and female rats, where HCA-SX was dissolved in water and administered by gavage at dose levels of 0.2, 2.0, and 5.0% of feed intake (equivalent to approximately 100, 1000, and 2500 mg/kg/day, resp.). The gavage dose volume was 5 mL/kg body weight. HCA-SX was administered by gavage rather than through feed as gavage administration most simulates the method of intake in humans, consumed over a relatively short period of time. The 0.2% HCA-SX supplementation is equivalent to the daily recommended dosage in humans, while 2.0 and 5.0% represent 10 and 25 times higher doses, respectively. Dose- and time-dependent effects of HCA-SX on body weight, hepatic and testicular lipid peroxidation and DNA fragmentation of mice over a period of 90 days were evaluated. HCA-SX of three doses significantly (*P* < 0.05) reduced body weight and feed intake in both male and female rats, but not water intake and lipid peroxidation. Moreover, no significant effects on liver and testis weight, hepatic and testicular DNA fragmentation morphology were observed in HCA-SX treated rats [24].

Further evaluation on the safety of HCA-SX was conducted by Shara et al. [25] where vital organ weights (including adrenal glands, brain, heart, kidneys, liver, prostate and seminal vesicles, spleen, testes and thymus in male rats, and adrenal glands, brain, heart, kidneys, liver, ovaries, spleen, thymus, and uterus in female rats) were assessed and correlated as a % of body weight and brain weight at 90 days of the treatment. No significant difference was detected between treatment groups. Besides, dose- and time-dependent effects of HCA-SX on hematology parameters (including WBC, RBC, hemoglobin, hematocrit and platelet count, and total serum protein and albumin) in male and female rats were examined. No significant difference was detected between treatment groups. Similarly, clinical chemistry analysis (alkaline phosphatase, blood urea nitrogen, creatinine, aspartate aminotransferase, alanine aminotransferase, cholesterol, total bilirubin, glucose, calcium, chloride, phosphorus, sodium, potassium, iron, total iron binding capacity, and iron/total iron bonding capacity) revealed no significant difference between treatment groups. Histopathology of different organs including adrenal glands, brain, epidedymes, esophagus, eyes, heart, intestine, kidney, liver, lymph nodes, lungs, mammary glands, ovary (females only), pancreas, pituitary, prostate, salivary glands, seminal vesicles, skin, spleen, stomach, testes (males only), thymus gland, thyroid gland, trachea, and urinary bladder of all treatment groups were assessed after 90 days of treatment. HCA-SX supplementation caused no significant morphological changes in the organs tested. Scattered minimal or mild histologic lesions observed in a number of organs were all randomly distributed in all groups and considered to be incidental findings commonly seen in rats. Besides, hemorrhage noted in brain appeared to be agonal or necropsy artifacts. The inflammatory lesions noted were in agreement with mild subclinical infections caused by *Mycoplasma *sp. The minimal hepatocyte vacuolation noted in HCA-SX groups were limited and not considered significant/treatment-related as one control animal had a similar lesion. Another change noted was within the glandular stomach where the mucosa of the glandular stomach of one animal was severely atrophied and mineralized. Besides, scattered minimal or mild foci of gastric gland dilation were also noted. No necrosis or inflammation was seen. These changes were noted in animals supplemented with HCA-SX as well as the untreated control group. The results obtained did not indicate the change being either more severe or more numerous in any particular dose group. In any case, the morphologically changes noted appeared to be minimal and not significant, thus indicating the safety and efficacy of HCA-SX in weight management [25].

Another study performed by Roy et al. [15] showed that none of the animals in their study exhibited early removal criteria such as self-mutilation, guarding, vocalization, hunched posture, inactivity, lethargy, rough hair coat, lack of righting reflex, weight loss of more than 20%, lesions, bleeding, and anorexia for >24 h. In addition, DNA microarray analysis showed that HCA supplementation did not affect vital genes associated with transcription of mitochondrial/nuclear protein and those essential for fundamental support of tissue. Taken together, dietary HCA-SX supplementation at a dose of 10 mg per kg body weight which corresponds to a 500 mg daily dose for an average person weighing 50 kg was safe.

### 2.5. Skin Irritation Studies

In another study, the potential systemic toxicity and local irritative potential of HCA-SX were evaluated using Albino rabbits. HCA-SX administration at a single dose of 500 mg/site was directly applied to shaved intact skin to assess the local dermal irritative potential. Each animal received a single, 4 h semioccluded exposure and application sites were evaluated at approximately 30–60 min and 24, 48, and 72 h after patch removal. Minimal irritation was noted in this study. Very slight erythema on a single animal was noted at the beginning of observation, but completely subsided by the end of day 1. All dose sites were stained yellow. No gross toxicological pathology (except for reddened application sites and accessory spleen for two rabbits each, and single occurrence of pale kidneys, mottled lungs, and hair loss for one rabbit each) was found on autopsy. HCA-SX was classified as nonirritating, as the primary irritation index was calculated to be 0 [12].

### 2.6. Eye Irritation Studies

HCA-SX was administrated by direct conjunctival instillation, a standard administration route for local ocular irritative potential assessment. HCA-SX at a dose of 54 mg/right eye was applied directly into the cupped lower conjunctival sac of the right (test) eye of six New Zealand white Albino rabbits. In this study, no death or significant changes in body weights (*P* < 0.05) was noted. Occular observation revealed that a small area of inflammatory exudate with enlarged blood vessels was present at the apex of the lower conjunctival sac for three rabbits on day 7. The inflammation completely subsided by the end of study for two animals, but not the other one. Positive iridal and conjunctival reactions were induced in all animals, but subsided within 48 h. The maximum average score of 15 (out of 110) was obtained, indicating HCA-SX possessed mild irritation on eye [12].

### 2.7. Reproduction and Developmental Studies

*G. cambogia*/HCA has been safely used in cooking and as weight-loss herbal supplements for many decades, but not without precedent that adverse effects had been reported. Saito et al. [26] investigated the dose-dependent ability of *G. cambogia* extract (containing 41.2 wt% of (−)-HCA, the ratio of free to lactone form was 36.6 to 63.4) in suppression of body fat accumulation and the safety of its high doses. Diets containing different levels of *G. cambogia* (equivalent to 0, 10, 51, 102, and 154 mmol HCA/kg diet) were fed to 6-week-old male Zucker obese rats for 92 or 93 days. Significant increases (*P* < 0.05) in ATP-citrate lyase activity and concentrations of liver glycogen, and reduction (*P* < 0.05) in plasma nonesterified fatty acid were detected in rats fed with 154 mmol HCA/kg diet. A dietary HCA level over 3.0 wt% (154 mmol HCA/kg diet) caused severe diarrhea in rat models. HCA administration reduced the testis weights by half in male Zucker obese rats. Histopathological examinations revealed marked testicular atrophy and impairment of spermatogenesis in the highest and second highest HCA groups. However, no significant differences in all the parameters in this study were observed in rats fed with 0, 10, and 51 mmol HCA/kg diet. Taken all together, the authors suggested that high dose of *G. cambogia* effectively suppresses fat accumulation in developing male Zucker obese rats, but was highly toxic to the testis.

In a continuation to the study conducted by Saito et al. [26], Kiyose et al. [27] investigated the cause of impaired spermatogenesis due to ingestion of *G. cambogia* powder (containing 41.2 wt% of (−)-HCA, the ratio of free to lactone form was 36.6 to 63.4) at 102 mmol/kg diet in young Fischer 344 male rats. By considering that 4,4-dimethyl-5*α*-cholesta-8,24-diene-3*β*-ol, a testis meiosis-activating sterole (T-MAS), was a specific intermediate product of cholesterol biosynthesis in testicular germ cells, the authors elucidated the relationship between impaired spermatogenesis and MAS production in rats testis, postingestion of *G. cambogia*. Histopathology examinations of rat testis showed that spermatogenesis was immature in all rats of both treatment and control group after 2 weeks of *G. cambogia* administration (six-week-old, sexually immature). After 4 weeks (eight-week-old, sexually mature), normal spermatogenesis was observed in control group, with abundance of elongation and elongated spermatids in all seminiferous tubules. On the contrary, there was a complete absence of spermatid elongation in the *G. cambogia* group, with some round spermatids being released in clusters instead. The concentrations of testosterone, luteinizing hormone (LH), follicle-stimulating hormone (FSH), and inhibin-B, the four hormones related to spermatogenesis, were measured. No significant difference (*P* < 0.05) in testosterone and LH concentrations were detected between groups. However, a significant reduction of inhibin-B concentration was detected in the *G. cambogia*-treated group, concurrently with an increase in FSH concentration, compared to the control group after 2 weeks of feeding. Similar results were obtained after 4 weeks of feeding, when the rats reached sexual maturation. Inhibin-B is an important marker of the function of Sertoli cells and spermatogenesis. The concomitant decrease in inhibin-B and increase in FSH concentrations indicated impaired spermatogenesis [28–30]. Therefore, the authors revealed that severe impairment of spermatogenesis occurred in rats administered (−)-HCA-containing *G. cambogia*, probably associated with a blockade of MAS substances accumulation.

With regards to the study conducted by Saito et al. [26], Burdock et al. [31] had raised several questions, such as toxicity associate with the form of (−)-HCA and the use of Zucker rats as a model in testicular toxicity evaluation. They suggested the possibility that the use of the atypical form of HCA containing 63% lactone resulted in toxicity. In addition, they indicated that Zucker rat may not be an appropriate model to evaluate testicular toxicity as obese male Zucker rat has a defect in testicular testosterone production [32]. The same question of whether the toxicity was possibly due to the atypical form of HCA containing 63% lactone could be applied on the study conducted by Kiyose et al. [27]. Therefore, the claim of HCA in affecting the functionality of testis is yet to be concluded.

Nevertheless, it would be more convincing to evaluate the effect of HCA consumption in fertility of both male and female models rather than only the testicular function to justify the effect of HCA on reproductive capacity. Pragmatic maternal observations indicated that maternal toxicity might occur due to reduced weight gain during pregnancy. Decrease in body weight of about 20% had been reported to possess adverse effects on fertility and reproduction in rats and mice [33]. In this regard, *G. cambogia*/HCA has been used by women for weight loss after delivery. However, Shara et al. [24, 25] reported that decrease in body weight in the range of 10-15% did not adversely affect weight and histopathology of both male and female reproductive organs of Sprague-Dawley rats. In their 90-day study, administration of HCA-SX at a dose of 5% of dietary intake resulted in 10–15% of weight loss in both male and female rats. Observations from human studies also demonstrated weight loss of approximately 10% following consumption of HCA-SX for a period of 4–12 weeks [14, 34, 35]. Hence, HCA is expected to cause no adverse effect on fertility and reproduction on the basis of weight loss.

A two-generation reproduction toxicity study was also conducted to evaluate the effects of HCA-SX on the reproductive systems of male and female rats, postnatal maturation and reproductive capacity of their offsprings, and possible cumulative effects through multiple generations. Rats were fed with diet containing 0, 1000, 3000, or 10,000 ppm of HCA-SX for a period of 10 weeks prior to mating, during mating, and across two generations, until their termination. No treatment-related adverse effects on reproductive performance in terms of fertility and mating, gestation, parturition, litter properties, lactation, sexual maturity, and development of offspring were observed during HCA-SX exposure of male and female rats from F_0_ and F_1_ generations. These results suggested that the “no observed adverse effect level (NOAEL)” of HCA-SX in both parental and offspring exceeded 10,000 ppm (equivalent to 1018 and 1524 mg/kg/day, in male and female rats, resp.) [36].

Several mechanism of action studies showed that HCA did not affect fatty acid synthesis in the fetus [37–39]. Jones and Ashton (1976) reported that HCA did not inhibit lipid synthesis in slices of fetal liver from guinea pigs, despite changes in fat synthesis and storage [37]. Besides, HCA did not affect fatty acid synthesis in explants of 18-day fetal lung tissue stimulated by the hormone dexamethasone, a drug known to stimulate the synthesis of fatty acids [38]. Another study conducted by Greenwood et al. [39] showed that HCA supplementation decreased feed intake and body weight of Zucker obese female rats, without affecting the percent of body fat and the fat cell size of these rats, as compared to the controls. These results suggested that HCA will not affect fatty acid synthesis in either the maternal animals or their offspring. In a continuation of the two-generation reproductive toxicity study, Deshmukh et al. [40] conducted a developmental toxicity study to evaluate the teratogenic potential of HCA-SX in Sprague-Dawley rats. In their teratology study, the rats were selected randomly postweaning from each F_2_ litter of the F_1_ generation from the two-generation reproductive toxicity study and allowed to grow up to 10 to 12 weeks of age before mating. Dietary exposure levels of 1000, 3000, and 10,000 ppm (equivalent to the dose levels of 103, 352, or 1240 mg/kg/day, resp.) were subjected indirectly to the male and female rats in HCA-SX treatment groups during lactation, and directly postweaning (4 weeks old) till they were terminated (including growth phase, mating period, and gestation). Maternal toxicity and effects on the developing embryo were evaluated throughout the gestation period until the 20th day of gestation. Apart from a slight (13%) lowering of maternal body weight gain in the group administered 10,000 ppm HCA-SX, no evidence of maternal toxicity, adverse effects on the parameters evaluated for the gravid uteri, external abnormalities in the fetuses, soft tissue abnormalities in the fetuses, or skeletal abnormalities in the fetuses was noted. The results suggested that HCA-SX (up to dose level of 1240 mg/kg/day) was not teratogenic to Sprague-Dawley rats. Considering the comprehensive reproductive and developmental studies reported on safety profile of HCA [24, 25, 36–40], it was strongly suggested that HCA consumption possessed no reproductive and developmental toxicity.

## 3. Clinical Toxicity

A total of 17 clinical studies with approximately 873 subjects were summarized to assess the effects of HCA and HCA-SX intake on human body weight and its safety for human consumption. Out of these studies, only 1 subject was reported itching around the mouth and 2 with headache and nausea. Taken all together, these studies provided sufficient qualitative and quantitative scientific evidence to report “no observed adverse effect level (NOAEL)” at levels up to 2800 mg/day, suggesting its safety in-use [41, 42]. In this section, we have analyzed the symptoms of adverse reactions reported in 15 clinical trials carried out in human subjects after the administration of *G. cambogia *extract (Table 1). There are 12 parallel, randomized, double-blind, placebo-controlled studies, involving 745 subjects [43–54], one parallel, randomized, single-blind, placebo-controlled study [55], three cross-over, randomized, double-blind, placebo-controlled trials [56–58], one cross-over, randomized, single-blind, placebo-controlled study [59], and one reexamination [50] of the data from two previous parallel, randomized, double-blind, placebo-controlled clinical trials [34, 60]. Out of 16, only nine of the clinical studies were performed with *G. cambogia *extract/HCA alone.

A number of hepatotoxicity cases associated with the consumption of hydroxycut had lead to the assumption that HCA is the primary causative agents to the hepatotoxicity [61–65]. Those products having adverse reactions were either polyherbal or multicomponent in nature. Furthermore, polyherbal dietary supplements reported with adverse effects either contain HCA in negligible amounts or no HCA at all. These polyherbal products contain up to 20 different ingredients, with only 8 out of the 14 marketed hydroxycut products contain HCA [66, 67], and in only two acute liver injuries associated with use of hydroxycut was HCA shown to be present in the product [61]. However, a single case report on adverse effects in *G. cambogia* extract/HCA-containing dietary supplement does not justify definitive attribution of causality in most cases. It is impossible to tell with certainty which ingredient(s) is responsible for the adverse effects reported in the various case reports. The majority of the case reports were insufficiently documented to make an informed judgment about a relationship between the use of *G. cambogia* extract/HCA or *G. cambogia* extract/HCA-containing dietary supplements and the adverse event in question. Mozersky et al. [68] also suggested in the Health Hazard Report on hydroxycut that the board did not know which type of ingredient(s) present in hydroxycut was the causative agent(s) of hepatotoxicity. In addition, there were no preclinical animal studies and clinical studies showing HCA consumption had direct adverse effects. The results obtained from various reports suggested a dosage of *G. cambogia* extract in clinical trials ranging from 1,500 to 4,667 mg/day (25 to 78 mg/kg/day), whereby their equivalent HCA dose ranging from 900 to 2,800 mg/day (15 to 47 mg/kg/day) is safe for human consumption [41, 69]. *G. cambogia* is available in capsule or tablet form with a maximum dose of 1,500 mg/day. A study conducted by Deshmukh et al. [40] determined the dietary dose levels of 1240 mg/kg/day as the NOAEL of HCA-SX.

## 4. Summary

Based on the results obtained in an array of toxicological and safety studies, a comprehensive safety profile on *G. cambogia* extract/HCA as dietary supplements for treating obesity has been established [41, 42, 66, 70] (Table 2). Cytotoxicity study [21], genotoxicity study [22, 23], acute toxicity studies (such as acute oral, acute dermal, primary dermal irritation, and primary eye irritation toxicity studies) [11, 12], sub-chronic 90-day safety study [15, 24, 25], two-generation reproductive and teratogenicity studies [24, 25, 36–40], and clinical studies on *G. cambogia* extract/HCA [43–58] support its safety demonstrating a wide margin of safety for human consumption. Recent animal and clinical toxicology studies have shown that *G. cambogia*/HCA is generally safe and is classified as NOAEL up to 1240 mg/kg/day [40]. In experimental animal studies at up to 233x the human equivalency dose of HCA (1500 mg/day of HCA), toxicological studies revealed no death, remarkable body weight changes, or gross necropsy findings in Albino rats [12]. Furthermore, the fact that *G. cambogia* extract has been widely used as an antiobesity herbal supplement for decades around the world without a birth defect or reproductive problem suggests that HCA is unlikely to cause reproductive or developmental toxicity. However, most randomized control trials (RCTs) have been conducted on small samples and mainly over a short term. None of them have shown whether the efficacy and safety of *G. cambogia* extract/HCA consumption persist beyond 12 weeks of intervention. Thus, more long term clinical trials or followups could be conducted, especially on consumers who have been taking HCA for a long period of time to add value to the NOAEL for long-term consumption.

## Figures and Tables

**Table 1:** Summary of clinical studies conducted to date on the results and safety record of HCA. Only the subjects who manage to complete the trial is counted in the table below.

Duration	Mode of trial	Formulation	Results	Safety	Conclusion	Reference
8 weeks	Parallel, randomized, double-blind, placebo control, 39 subjects	1500 mg *G. cambogia *+ 300 *μ*g chromium picolinate/day	No significant effect between groups	Itching around mouth in both treatment and placebo groups.	None toxic	[43]

8 weeks	Parallel, randomized, double-blind, placebo control, 35 subjects	1500 mg *G. cambogia *before meal/day	No changes in blood glutamic oxaloacetic transaminase (SGOT), glutamic pyruvic transaminase (SGPT) and glucose.	Headaches and nausea in both treatment (2) and placebo (1) groups	None toxic	[44]

4 weeks	Parallel, randomized, double-blind, placebo control, 144 subjects	55 mg *G. cambogia *+ 19 mg chromium + 240 mg chitosan/day	Treated group possessed significant weight loss, lower TC, LDL and higher HDL as compared to placebo	Headaches and nausea in both treatment (2) and placebo (1) groups	None toxic	[45]

12 weeks	Parallel, randomized, double blind, placebo control, 84 subjects	3000 mg *G. cambogia* (50% HCA)/day	No significant effect between groups	Intestinal disorders, headache, or upper airway symptoms in both treatment and placebo groups	None toxic	[46]

6 weeks	Parallel, randomized, double blind, placebo control, 18 subjects	750 mg *G. cambogia *+ 750 mg calcium + 750 mg guggulsterone + 750 mg L-tyrosine/day	No significant effect between groups	Not reported	—	[47]

12 weeks	Parallel, randomized, double blind, placebo control, 33 subjects	300 mg *G. cambogia* + 1200 mg *Phaseolus vulgaris *+ 1200 mg inulin/day	Better weight lose in treated group	Not reported	—	[48]

12 weeks	Parallel, randomized, double blind, placebo control, 89 subjects	2400 mg *G. cambogia*/day	Better weight lose in treated group	Not reported	—	[49]

8 weeks	Parallel, randomized, double-blind, placebo-control, 82 moderate obsese subjects	2800 mg HCA; 4667 mg of HCA-SX in combination with niacin-bound chromium and standardized *Gymnema sylvestre* extract/day	Significant weight loss, reduction in BMI, increased fat oxidation, favorable lipid profile, reduction in circulating plasma leptin levels, increase in serum serotonin levels, and decreased appetite as determined by reductions in food intake were detected in HCA-SX treatment group, and to a greater extent the combination of the 3 ingredients	No serious adverse effects were detected, except several minor adverse effects such as leg cramps, heartburn, diarrhea, gas, increased appetite, headaches, stomach burn, and menstrual disorders	None toxic	[50]

12 weeks	Parallel, randomized, double blind, placebo control, 98 subjects	*G. cambogia* + kidney bean pods + chromium yeast	Better weight lose in treated group	More gastrointestinal symptoms in treated group.	None toxic	[51]

12 weeks	Parallel, randomized, double blind, placebo control, 44 subjects	1,667.3 mg of *G. cambogia* extract/day (1,000 mg HCA/day)	No significant effect on TG between treatment and placebo group. Answer to the concept of potential spermatogenesis impair [27]	No significant reproductive toxicity on serum testosterone, estrone, and estradiol levels, hematology, serum triacylglycerol, and serum clinical pathology parameters	None toxic	[52]

12 weeks	Parallel, randomized, double blind, placebo control, 58 subjects	2400 mg *G. cambogia *+ 1500 mg *Amorphophallus konjac*/day	No significant effect between groups	No significant difference between treatment and placebo groups	None toxic	[53]

2 weeks × 3 times	Parallel, randomized, double blind, placebo control, 21 subjects	500 mg HCA + 300 medium chain TG/day	No significant effect between groups	Not reported	—	[54]

8 weeks	Parallel, randomized, single blind, placebo control, 40 subjects	1000 mg of HCA/day	Reduction of visceral fat area and visceral fat area/subcutaneous fat area	No significant difference in hematological parameters (white blood cells, red blood cells, hemoglobin, hematocrit and platelets) and clinical chemistry parameters (SGPT, SGOT, c glutamyl transpeptidase, lactate dehydrogenase, blood urea nitrogen, creatinine, glucose, insulin, acetoacetic acid, 3-hydroxybutyric acid, and total ketone bodies) between groups	None toxic	[55]

10 days	Cross-over, randomized, placebo control, 44 subjects	1000, 2000, 3000 and 4000 mg* G. cambogia*/day		No significant different in hematology and clinical chemistry analysis before and after treatment no unusual electrocardiographic effects.	NOAEL > 4 g HCA	[56]

5 hours	Cross-over, randomized, double blind, placebo control, 20 subjects	extracts of, *G*.* cambogia*, green tea, caffeine, and yerba mate		No unusual electrocardiographic effects.		[57]

??	Cross-over, randomized, double blind, placebo control	5600 mg HCA/day	??	Yet to be published	??	[58]

2 weeks	Cross-over, randomized, single blind, placebo control, 24 subjects	900 mg HCA/day	Decreased energy intake	No adverse effect	None toxic	[59]

**Table 2:** Summary on the advantages, disadvantages, benefits, and pitfall of up-to-date *in vitro*, *in vivo *and clinical toxicology studies on *Garcinia*/HCA.

Methodology	Study target	Summary	Advantages	Disadvantages	Benefits	Pitfall of experiment
*In vitro * cytotoxicity	3T3 fibroblast [21]	*G. indica *was cytotoxic on 3T3	Rapid test	Not fully representative compared to animal/human subject.	First line screening	Poor methodology, only Balb/c 3T3 was screened.

*In vitro *genotoxicity ~Ames test ~Chromosomal aberration test	~*Salmonella typhimurium*i, ~Chinese hamster ovarian cell [22]	HCA-SX did not induced mutagenic activity	Rapid test	Not fully representative compared to animal/human subject.	First line screening	

*In vivo *genotoxicity Micronucleus test	8 weeks old ICR mice [22]	Micronucleated polychromatic erythrocytes in bone marrow cell	Better representation than *in vitro *cell line study	Variation among animal.	Preclinical screening	i.p. injection with DMSO as vehicle not suitable; no prior i.p. LD_50_ predetermination; 12,500 *μ*mol/kg exceed the highest dose, poor statistic analysis [23].

*In vivo *acute toxicity	Albino rat [12]	HCA SX LD_50_ > 5 g/kg body weight	High dosage (233X higher than maximum dose of 1.5 g/day in human)	Single administration.	Understand acute toxic effect at high concentration	Only LD_50_, gross necropsy and body weight were recorded. No blood biochemical profiling and full blood count.

*In vivo *subchronic	Rat [24, 25]	HCA-SX reduced body weight, feed intake but no effect on other parameters.	Experiment was design to represent actual recommended dosage.	—	Good reference to support the entry of clinical studies.	—

*In vivo *skin irritation	Albino rabbit [12]	HCA-SX was none irritating with primary irritation index = 0.	More representative than *in vitro *test.	Single exposure.		This study only tested the irritative potential with single exposure.

*In vivo *eye irritation	Albino rabbit [12]	HCA-SX was mild irritant on eye.	More representative than *in vitro *test.	—	HCA-SX is an oral supplement. Results for *in vivo *skin and eye irritation can help to strengthen the MSDS.	—

*In vivo *reproduction toxicity	Rat [36, 40]	HCA-SX did not affect the postnatal maturation, reproductive capacity.	“No observed adverse effect level” of HCA-SX higher than 1.5 mg/kg/day was determined in both parental, offspring generation and HCA-SX was not teratogenic.	—	Good reference to support that HCA was none toxic effect against reproductive system.	—

*In vivo *reproduction toxicity	Zucker obese rats [26, 27]	*G. cambogia* powder (containing 41.2 wt% of (−)-HCA, the ratio of free to lactone form is 36.6 to 63.4) impaired spermatogenesis	—	—	—	Zucker rat is not suitable in this study since it has a defect in testicular testosterone production. HCA used in this experiment contains high lactone that may contributed to it the impairment of spermatogenesis [31]

Clinical studies (as stated in Table 1)	873 subjects	*Garcinia*/HCA is generally none toxic with NOAEL > 4 g HCA	*Garcinia*/HCA was recorded as none toxic up to 12 weeks consumption.	None of the studies recorded the use of *Garcinia*/HCA for more than 12 weeks.	*Garcinia*/HCA is generally safe to consume up to 3 months.	Continue monitoring on the consumers who take *Garcinia*/HCA for more than 3 months can strengthen the knowledge of long term safety assessment of *Garcinia*/HCA.
